# EAST MEETS WEST: IMPROVING HEALTH AND LONG-TERM CARE FOR OLDER ADULTS IN THE U.S. AND CHINA

**DOI:** 10.1093/geroni/igz038.914

**Published:** 2019-11-08

**Authors:** Bei Wu, Jie Hua Lu

**Affiliations:** 1 New York University, New York, New York, United States; 2 Department of Sociology, Peking University, Beijing, Beijing, China

## Abstract

As the number of older adults in the U.S. and China continues to increase, promoting healthy aging is essential for individuals, family, and society. Both countries face many similar issues due to their aging populations, including prolonging healthy life expectancy and providing quality of care. However, the change in demographics brings with it unique challenges for both the U.S and China. This forum invites scholars and researchers from these two countries to share their knowledge and insights on promoting healthy aging and improving care for older adults. This forum includes five presentations and one-panel discussion. Two presentations will focus on long-term care (LTC) in China, one is to forecast the needs of LTC in the next five decades, and the other is to evaluate the current LTC needs and discuss LTC policy. Using the data from the Health and Retirement Study, the third presentation aims to re-conceptualize spousal caregiving as a dyad-level phenomenon and provides a dynamic view of the spousal caregiving experiences. The last two presentations will focus on promoting healthy aging through clinical interventions. The fourth one is to evaluate the effectiveness of adaptive computer-based cognitive training among community-dwelling older adults in China. The last presentation provides some examples of using pragmatic clinical trials to improve the care of older adults in skilled nursing facilities in the U.S. After the five presentations, the three panelists will provide feedback to the presentations and share their views on healthy aging with the audience.

